# Increased malaria transmission around irrigation schemes in Ethiopia and the potential of canal water management for malaria vector control

**DOI:** 10.1186/1475-2875-13-360

**Published:** 2014-09-13

**Authors:** Solomon Kibret, G Glenn Wilson, Habte Tekie, Beyene Petros

**Affiliations:** Ecosystem Management, School of Environmental and Rural Science, University of New England, Armidale, NSW 2351 Australia; Department of Zoological Sciences, Addis Ababa University, PO Box 1176, Addis, Ababa Ethiopia; Department Microbial, Cellular and Molecular Biology, Addis Ababa University, PO Box 1176, Addis Ababa, Ethiopia

**Keywords:** Malaria, Irrigation, Canal water management, Anopheline mosquito breeding, Ethiopia

## Abstract

**Background:**

Irrigation schemes have been blamed for the increase in malaria in many parts of sub-Saharan Africa. However, proper water management could help mitigate malaria around irrigation schemes in this region. This study investigates the link between irrigation and malaria in Central Ethiopia.

**Methods:**

Larval and adult mosquitoes were collected fortnightly between November 2009 and October 2010 from two irrigated and two non-irrigated (control) villages in the Ziway area, Central Ethiopia. Daily canal water releases were recorded during the study period and bi-weekly correlation analysis was done to determine relationships between canal water releases and larval/adult vector densities. Blood meal sources (bovine *vs* human) and malaria sporozoite infection were tested using enzyme-linked immunosorbent assay (ELISA). Monthly malaria data were also collected from central health centre of the study villages.

**Results:**

Monthly malaria incidence was over six-fold higher in the irrigated villages than the non-irrigated villages. The number of anopheline breeding habitats was 3.6 times higher in the irrigated villages than the non-irrigated villages and the most common *Anopheles* mosquito breeding habitats were waterlogged field puddles, leakage pools from irrigation canals and poorly functioning irrigation canals. Larval and adult anopheline densities were seven- and nine-fold higher in the irrigated villages than in the non-irrigated villages, respectively, during the study period. *Anopheles arabiensis* was the predominant species in the study area. *Plasmodium falciparum* sporozoite rates of *An. arabiensis* and *Anopheles pharoensis* were significantly higher in the irrigated villages than the non-irrigated villages. The annual entomological inoculation rate (EIR) calculated for the irrigated and non-irrigated villages were 34.8 and 0.25 *P. falciparum* infective bites per person per year, respectively. A strong positive correlation was found between bi-weekly anopheline larval density and canal water releases. Similarly, there was a strong positive correlation between bi-weekly vector density and canal water releases lagged by two weeks. Furthermore, monthly malaria incidence was strongly correlated with monthly vector density lagged by a month in the irrigated villages.

**Conclusion:**

The present study revealed that the irrigation schemes resulted in intensified malaria transmission due to poor canal water management. Proper canal water management could reduce vector abundance and malaria transmission in the irrigated villages.

## Background

Sub-Saharan Africa uses only 3.9% of its renewable water resources, and only 6.3% of its arable land is irrigated [1]. The region is long known for malnutrition and it has been predicted that climate change threatens to increase malnutrition in the region by 14 million by 2020, with staple food production in many sub-Saharan countries falling by more than 25% [2]. Alarmed by these threats, several large- and small-scale irrigation schemes are under construction across the region with the goal of ensuring food security and alleviating poverty [3]. However, the negative impact of these emerging irrigation schemes on vector-borne disease, especially malaria – a disease that causes between 300 and 500 million infections and some 655,000 deaths globally each year [4] – has been a huge concern [5].

Development of irrigation schemes in sub-Saharan Africa has been blamed for the increase of malaria risk through creating favourable breeding sites for malaria vector mosquitoes [5–9]. However, water resources development also brings opportunities of cost-effective vector control measures through proper water management to create conditions less favourable for mosquito vector breeding [10–14].

Nevertheless, most studies assessing water management options for malaria vector control in sub-Saharan Africa focus on rice irrigation [7, 11, 14–18] with limited information on irrigation on other crops. Thus, it is crucial to assess the impact of using proper water management for malaria vector control around different crop irrigation schemes. Understanding how water management could be applied for malaria vector control in different irrigation practices will help devise water management options to mitigate malaria around irrigation schemes in Africa. The present study aims to assess the relationship between irrigation practices and malaria transmission and evaluate water management options to mitigate malaria transmission around irrigation schemes in Central Ethiopia.

## Methods

### Study area

The study was conducted in the Ziway area (8^o^00’N, 38^o^40’E) located 165 km south of Addis Ababa, the capital of Ethiopia, in the middle of the Ethiopian Rift Valley in Central Ethiopia. The area lies at 1,650 m above sea level with a semi-arid environment. It receives between 700 and 800 mm of annual rainfall, with the main rains from June to September and short rains in April and May (National Meteorological Agency, unpublished data). The mean annual temperature is 20 °C. The Ziway area is known for its irrigation practice, which covers an irrigated land of some 205 sq km. The source of water for irrigation is Lake Ziway, located 10-15 km from the irrigated farmland. The water is pumped using three engine-operated pumps feeding three primary earthly canals. The irrigated area extends to some 65 ha of land, mainly producing maize, corn and onions.

Two irrigated (Abime and Kontella) and two non-irrigated (Washigulla and Telanto) villages were selected for mosquito collection. The irrigated villages, situated within 1 km radius from the irrigated farms, had a population of 5,342 in 2009. A previous study showed that households using irrigation earn about a five-times higher income (US$550 per household per month) compared to farming households without irrigation (US$105 per household per month) [19]. However, despite significant income differences, household expenditure on anti-malarial measures (e.g. bed nets and personal protections) was found to be comparable and generally low in both settings [19]. The explanation for this could be the presence of low knowledge, attitude and practice of malaria vector control measures in the irrigated villages [19]. The non-irrigated (control) villages, with a total population of 4,898, are located some 20-25 km from the irrigation scheme but with similar agrarian socio-economic standards except irrigation practice. In both irrigated and non-irrigated villages, housing is mud walls with thatched or iron roofing. Cattle rearing and mud brick making are common practices in all study villages. Access to health care was comparable in irrigated and non-irrigated study villages – on average one health post for 2,048 and 2,107 people, respectively (District Health Office, unpublished data).

Malaria is the leading public health challenge with an unstable/seasonal trend, peaking between September and December following the major wet season months (June-August) [9]. *Plasmodium falciparum* is the predominant malaria parasite, causing 70% of malaria infections, followed by *Plasmodium vivax* [9, 20]. *Anopheles arabiensis* is the primary malaria vector species while *Anopheles pharoensis* plays secondary role [9].

### Retrospective clinical malaria data collection

To assess the impact of irrigation on malaria risk, retrospective monthly laboratory-confirmed malaria data were collected from the Ziway Health Centre during the study period (November 2009 – October 2010). A malaria dataset was sorted by village, and type of malaria parasite as confirmed by microscopy. Monthly malaria incidence (cases per 1,000 population per month) was calculated for each village to determine the level of malaria transmission in each village across months of the year.

### Mosquito sampling

Larval and adult mosquitoes were collected fortnightly from the irrigated and non-irrigated study villages between November 2009 and October 2010. At each larval sampling, all available potential mosquito breeding habitats such as irrigation canals, canal leakage pools (i.e., pools formed from leaking main canals), irrigated field paddies (water-logging in the field due to over-irrigation and poor drainage canals), mud-brick-making pits, rain pools and other man-made pools, such as water-holding wells were surveyed within a 1 km radius of each study village using standard dippers (350 ml) [21]. The surface area of each potential mosquito-breeding site was estimated in square metre (sq m) and sampling was made at a rate of six dips per sq m (four dips at the margin and two from the middle). Accordingly, sampling was undertaken proportional to the water surface area [9]. Larval anophelines sampled from each type of breeding habitat were kept in separate vials by direct pipetting. Larvae were killed by gently heating and preserved in 70% alcohol for later species identification. In the laboratory, preserved anopheline larval samples were counted and individually mounted on microscope slides using gum chloral for species identification based on morphological characteristics [22].

Adult mosquitoes were sampled fortnightly using CDC light traps (Model 512; J W Hock Co, Atlanta, USA) between November 2009 and October 2010. A total of 12 light traps were installed in each study village and operated from 18.00 to 06.30 hours during each sampling night. Half of the light traps were operated indoors and the other half installed outdoors in each of the study villages. Houses were randomly selected for light trap mosquito sampling, but the same houses were used throughout the study period. Each indoor light trap was hung on a wall, with the bulb about 45 cm above the head of a person sleeping under an untreated bed net [23]. Outdoor light traps were hung on trees at close proximity (~50 to 100 m) to open cattle enclosures where some individuals spent the evening. In the field laboratory, anopheline mosquitoes were sorted from adult mosquito captures, counted and further identified into species using morphological characteristics [24]. All female anopheline specimens were kept at room temperature (19-22°C) at the Addis Ababa University, Biomedical Science Laboratory, for later processing.

### Mosquito processing

The head-thorax portion of each dried female anopheline was subjected to circumsporozoite malaria parasite antigens (*P. falciparum* and *P. vivax*) detection using enzyme-linked immunosorbent assay (ELISA) [25]. The abdomen portion of fed anophelines were tested for blood meal sources (human *vs* bovine) using the direct ELISA technique [26].

### Environmental variables

Daily canal water releases were recorded from the three primary canals using water flow measuring probe (Global FP111 Flow Probe, Geo Scientific Ltd, Canada). The water probe was attached to a digital recorder to download the data to a computer, and the data were then exported to Microsoft Excel for analysis. Mean bi-weekly canal water discharges were calculated to determine the association with bi-weekly mosquito larval and adult densities. The relationship between bi-weekly vector densities and bi-weekly canal water discharges lagged by 2 weeks was assessed taking into consideration duration of larval development.

### Statistical analysis

Monthly malaria incidence was calculated as the number of laboratory-confirmed malaria cases in a given month among 1,000 population [27]. *Anopheles* larval density was expressed as the mean number of anopheline larvae per sq m, while adult mosquito density were expressed as the mean number of adult mosquitoes per light trap per night. Malaria incidence, larval and adult mosquito densities were compared between the irrigated and non-irrigated villages using Wilcoxon Signed Ranks Test [28].

The sporozoite infection rate of malaria vector species was calculated as the proportion of mosquitoes positive for *Plasmodium* sporozoites in the total number of mosquitoes of a species tested by ELISA. The difference in sporozoite rates between the irrigated and non-irrigated villages was analysed by Chi-square test. Man-biting rates were derived from light trap catches (i.e., adult anopheline density divided by a conversion factor 1.5 [23]. A study in northern Ethiopia reported that light traps are 1.5-times (95% CI = 1.2–1.8) more efficient than human landing catches [23]. The sporozoite rate was then multiplied by man-biting rate to determine entomological inoculation rates (EIR). The difference in sporozoite infection rates and annual EIR between the irrigated and non-irrigated villages was analysed by Chi-square test. The human blood index (HBI) for each *Anopheles* species was calculated as the proportion of samples positive for human blood from the total blood meals of each species tested by blood meal ELISA. Pearson’s correlation was employed to test the relationship between bi-weekly canal water releases and bi-weekly anopheline larval/adult densities in the irrigated villages. A similar statistical test was applied to determine the relationship between monthly adult vector density and malaria incidence lagged by one month – allowing for mosquito development and the *Plasmodium* incubation period. The level of significance was determined at 0.05. All analyses were done using Microsoft Excel 2003 and the statistical software, SPSS version 13 (SPSS Inc, Chicago, IL, USA).

## Results

### Malaria incidence

**Figure 1:**
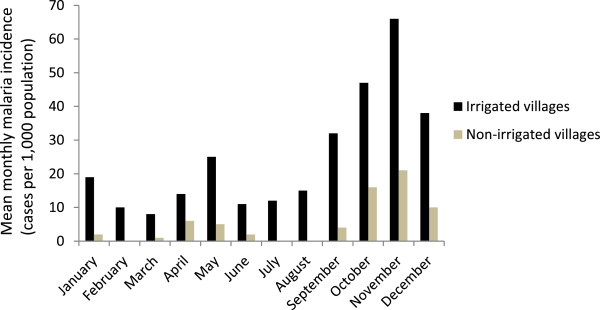
**Monthly malaria incidence (cases per 1,000 population) in the irrigated and non-irrigated villages in the Ziway area, Ethiopia, in 2010.**

The mean monthly malaria incidence (cases per 1,000 population) was significantly higher in the irrigated villages (33.7; 95% CI = 16.2-51.2; z = -2.431; *P* < 0.001) compared to the non-irrigated villages (5.6; 95% CI = 0.0-11.2) (Figure 1). In the irrigated villages, bimodal peak in malaria transmission was observed between September and November, and between April and June. The data showed that malaria transmission occurred across all months of the year in the irrigated villages, unlike the non-irrigated villages where transmission occurred mainly during the post-rainy months (September-November) with few or no cases in the dry season. Overall, monthly malaria incidence was over six-fold higher in the irrigated villages than the non-irrigated villages during the study period.

### Mosquito breeding and larval abundance

During the study period, a total of six types of mosquito-breeding habitats (i.e., brick-making pits, rain pools, non-functioning canal pools, leakage pools from irrigation canals and agricultural field puddles) were identified (Table 1). While all six types of mosquito-breeding habitats existed in the irrigated villages, only two (brick-making pits and rain pools) were found in the non-irrigated villages. The number of potential mosquito breeding sites was two times higher in the irrigated villages (n = 298) than non-irrigated villages (n = 143) during the study period. Of these, 53.7% (n = 160) and 34.5% (n = 45) were found positive for larval anopheline mosquitoes in the irrigated and non-irrigated villages, respectively. Agricultural field paddies and leakage pools from irrigation canals contributed over 75% of larval anopheline-breeding habitats in the irrigated villages while rain pools accounted for over half of the total larval breeding sites in the non-irrigated villages. Overall, there were 3.6 times more anopheline-breeding sites in the irrigated villages than the non-irrigated villages during the study period. Moreover, a total of 68 sq m of larval anopheline breeding water body was evident in the irrigated villages, compared to only 12 sq m in the non-irrigated villages during the study period.

**Table 1:** **Summary of potential and anopheline larval positive breeding sites in the irrigated and non-irrigated study villages in the Ziway area, Ethiopia, between November 2009 and October 2010**

Type of larval habitat	Irrigated villages			Non-irrigated villages		
	No potential breeding habitats (%)	No positive breeding sites (%)	Percentage of positive breeding sites	No potential breeding sites (%)	No positive breeding sites (%)	Percentage of positive breeding sites
Brick-making pits	20 (6.7)	9 (5.6)	45.0%	56 (39.2)	21 (46.7)	37.5%
Rain pools	41 (13.8)	20 (12.5)	48.8%	87 (60.8)	24 (53.3)	27.6%
Non-functioning canal pools	22 (7.4)	10 (6.3)	54.4%	-#	-	
Leakage pools from irrigation canals	55 (18.5)	25 (15.6)	45.4%	-	-	
Agricultural field puddles	160 (53.7)	96 (60.0)	60.0%	-	-	
Total	298 (100)	160 (100)	53.7%	143 (100)	45 (100)	31.5%

# - these breeding habitats did not exist.

A total of 2,959 larval anophelines comprising *An. arabiensis*, *An. pharoensis*, *Anopheles coustani*, and *Anopheles funestus* were collected during the study period (Table 2). Of these, the majority (85.5%; n = 2,531) were collected from the irrigated villages while the remaining 14.5% (n = 428) were from non-irrigated villages. *Anopheles arabiensis* was the most common species in all study villages. Larvae of this species were predominantly collected from irrigation-associated breeding sites. *Anopheles pharoensis* was the second-most abundant species predominantly occurring in agricultural field puddles and leakage pools from irrigation canals. Similarly, larvae of *An. coustani* and *An. funestus* were also found in these breeding habitats. In the non-irrigated villages, *An. arabiensis* was the dominant species, whose larvae were commonly found in rain pools, followed by *An. pharoensis* and *An. coustani*. No *An. funestus* larvae were found in the non-irrigated villages. Overall, the irrigation scheme provided considerable breeding habitats for malaria vector mosquitoes.

**Table 2:** **Summary of species composition and density of anopheline larvae in the irrigated and non-irrigated villages in the Ziway area, Ethiopia, between November 2009 and October 2010**

Types of breeding habitats	Mean larval density ^a^	Type of mosquito larval habitat				
		***An. arabiensis***	***An. pharoensis***	***An. coustani***	***An. funestus***	Total
		n (%)	n (%)	n (%)	n (%)	n (%)
**Irrigated villages**						
Brick-making pits	3.2	20 (1.5)	16 (1.9)	16 (5.1)	0 (0.0)	52 (2.0)
Leakage pools from irrigation canal	46.5	319 (24.5)	280 (32.9)	67 (21.2)	19 (29.2)	685 (27.1)
Non-functioning canal pools	29.3	139 (10.7)	57 (6.7)	41 (13.0)	0 (0.0)	237 (9.4)
Rain pools	12.4	292 (22.5)	130 (15.3)	63 (19.9)	0 (0.0)	485 (19.2)
Agricultural field puddles	41.9	530 (40.8)	367 (43.2)	129 (40.8)	46 (70.8)	1,072 (42.3)
Total (% from row total)	38.0	1,300 (51.3)	850 (33.6)	316 (12.5)	65 (2.6)	2,531 (100)
**Non-irrigated villages**						
Brick-making pits	4.1	56 (20.6)	9 (11.5)	8 (10.3)	-*	73 (17.1)
Leakage pools from irrigation canal^#^	-	-	-	-	-	-
Non-functioning canal pools^#^	-	-	-	-	-	-
Rain pools	9.3	216 (79.4)	69 (88.5)	70 (89.7)	-	355 (82.9)
Agricultural field puddles^#^	-	-	-	-	-	-
Total (% from row total)	6.7	272 (63.6)	78 (18.2)	78 (18.2)	-	428 (100)
Grand total (% from row total)	NA	1572 (53.1)	928 (31.4)	394 (13.3)	65 (2.2)	2,959 (100)

^a^mean larval density refers to no. anopheline larvae per sq m.

* - shows this mosquito species was not found in either larval or adult form in these villages.

^#^ - these mosquito breeding habitats were absent in the non-irrigated villages.

NA – not applicable.

**Figure 2:**
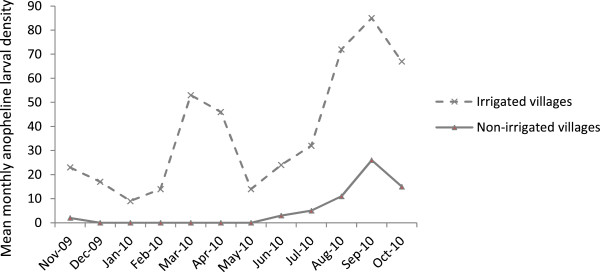
**Monthly trend of anopheline larval density (no. larvae per sq m) in the irrigated and non-irrigated villages in the Ziway area, Ethiopia, between November 2009 and October 2010.**

Monthly mean *Anopheles* larval density (mean number of larvae per sq m) varied considerably over the study period (Figure 2). A seven-fold higher anopheline larval density was evident in the irrigated villages (mean larval density = 38.0; 95% CI = 21.0-55.0; z = -4.10; *P* <0.001) as compared to the non-irrigated villages (6.7; 95% CI = 2.1-11.3) during the study period. In the irrigated villages, monthly anopheline larval densities were generally higher towards the end of the main rain season (July and September) with a peak in September and a shorter peak in the dry season in March. A similar pattern of monthly anopheline larval density fluctuation was observed in the non-irrigated villages, although larvae disappeared here during the dry season months.

### Adult anopheline abundance

A total of 5,852 adult anophelines were collected using CDC light traps in the study villages (Table 3). Of these, the majority (92%) were collected from the irrigated villages. All four *Anopheles* species identified in the larval samples were also present in adult collections. *Anopheles arabiensis* was the predominant species in both irrigated and non-irrigated villages followed by *An. pharoensis* and *An. coustani*. Adults of *An. funestus* were only found in the irrigated villages. The density of adult anophelines (mean number of anophelines per light trap per night) was significantly higher (*P* < 0.001) in the irrigated villages (mean = 19.2 anophelines/trap/night; 95% CI = 6.1-32.3) than the non-irrigated villages (1.9; 95% CI = 0.3-3.6) throughout the study period. Adult *An. arabiensis* densities were significantly higher indoors than outdoors while the other *Anopheles* species predominantly occurred outdoors.

**Table 3:** **Total number of adult anopheline collected using CDC light traps, and indoor and outdoor adult anopheline densities (mean number adult anophelines per light trap per night) in the irrigated and non-irrigated villages in the Ziway area, Ethiopia, between November 2009 and October 2010**

	***An. arabiensis***	***An. pharoensis***	***An. coustani***	***An. funestus***	Total
Irrigated villages					
N (%)*	2,945 (55)	1,678 (31)	569 (11)	165 (3)	5,357 (100)
No. collected indoors (density)	1,105 (15.33)	698 (5.82)	217 (1.81)	14 (0.12)	2,034 (23.08)
No. collected outdoors (density^#^)	1,840 (9.21)	980 (8.17)	352 (2.93)	151 (1.26)	3,323 (21.57)
Non-irrigated villages					
N (%)	321 (65)	105 (21)	69 (11)	0	495 (100)
No. collected indoors (density)	233 (1.94)	31 (0.26)	22 (0.18)	0.00	286 (2.38)
No. collected outdoors (density^#^)	88 (0.73)	74 (0.62)	47 (0.39)	0.00	209 (1.74)

*number of adult anophelines collected during the study period (and percentage from row totals).

^#^density refers to mean no. anophelines per trap per night.

**Figure 3:**
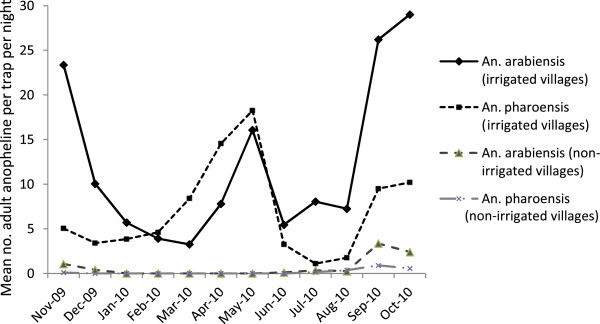
**Monthly malaria vector density (mean no. of adult anopheline per light trap per night) in the irrigated and non-irrigated villages in the Ziway area, Ethiopia, between November 2009 and October 2010.**

The monthly densities of *An. arabiensis* increased remarkably immediately after the long rainy season, between September and October (Figure 3). In contrast, the densities of *An. pharoensis* increased during the short rainy season with a peak in May. Overall, the densities of malaria vector mosquitoes were substantially higher in the irrigated villages than in the non-irrigated villages throughout the study period.

### Source of blood meal and entomological inoculation rate

ELISA bioassay results showed that *An. arabiensis* was the predominant human-blood feeding species with high HBI both in the irrigated (80%) and non-irrigated villages (73%) (Table 4). *Anopheles pharoensis* showed a slight preference for human blood (63.6%) over bovines (49.5%) in the irrigated villages, while it fed ambivalently on both humans (59.2%) and bovines (53.5%) in the non-irrigated villages. However, the HBI of *An. arabiensis* (*X*^2^ = 2.12; degree of freedom [df] = 1; *P* > 0.05) and *An. pharoensis* (*X*^2^ = 3.42; df = 1; *P* > 0.05) was not significantly higher in the irrigated villages than the non-irrigated villages. *Anopheles coustani* primarily fed on bovines in both irrigated (77.6%) and non-irrigated (72.6%) villages. Similarly, *An. funestus* primarily fed on bovines in the irrigated villages.

**Table 4:** **Sources of blood meal in female anophelines in the irrigated and non-irrigated villages in the Ziway area, Ethiopia, between November 2009 and October 2010**

	***An. arabiensis***	***An. pharoensis***	***An. coustani***	***An. funestus***
**Irrigated villages**				
Number tested^a^	2,101	992	215	58
Positive for human blood (%)	1,678 (80.0)	631 (63.6)	107 (49.8)	21 (36.2)
Positive for bovine blood (%)	593 (28.2)	491 (49.5)	156 (72.6)	45 (77.6)
Unidentified (%)^b^	16 (0.8)	21 (2.1)	9 (4.2)	2 (3.4)
**Non-irrigated villages**				
Number tested	234	71	29	-^c^
Positive for human blood (%)	171 (73.1)	42 (59.2)	11 (37.9)	
Positive for bovine blood (%)	56 (23.9)	38 (53.5)	23 (79.3)	-
Unidentified (%)	13 (5.6)	5 (7.0)	4 (13.8)	-

^a^Samples that were positive for both human blood and bovine blood were included in both categories. Hence the total in each row could be more than 100%.

^b^Unidentified blood was neither from humans nor from cattle.

^c^No *An. funestus* were collected in the non-irrigated villages.

*Plasmodium falciparum* sporozoites were detected in *An. arabiensis* (1.67%) and *An. pharoensis* (0.81%) specimens from the irrigated villages while only one *An. arabiensis* (0.43%) was found to be positive for malaria sporozoites in the non-irrigated villages during the study period (Table 5). In the irrigated villages, *P. falciparum*-positive *An. arabiensis* specimens were collected in May (n = 2), April (n = 3), September (n = 16), October (n = 9), November (n = 4), December (n = 1), indicating active malaria transmission both in dry and wet seasons, while only one *An. arabiensis* specimen in the non-irrigated villages was found to be positive in September. The annual EIR calculated for the irrigated villages were 27.3 and 7.5 *P. falciparum* infective bites per person per year by *An. arabiensis* and *An. pharoensis*, respectively. In the non-irrigated villages, the annual EIR for *An. arabiensis* was 0.25 *P. falciparum* infective bites per person per year. The difference in EIR between irrigated and non-irrigated villages was significant (*X*^2^ = 12.31; df = 1; *P* < 0.05). No *P. vivax* sporpzoites were detected from any of the samples tested in the irrigated and non-irrigated villages.

**Table 5:** ***Plasmodium falciparum*** **sporozoite rates in four** ***Anopheles*** **species collected from the irrigated and non-irrigated villages in the Ziway area, Ethiopia, between November 2009 and October 2010**

	***An. arabiensis***	***An. pharoensis***	***An. coustani***	***An. funestus***
**Irrigated villages**				
Number tested	2101	992	215	58
Number positive (%)	35 (1.67)	8 (0.81)	0 (0.0)	0 (0.0)
**Non-irrigated villages**				
Number tested	234	71	29	-*
Number positive (%)	1 (0.43)	0 (0.0)	0 (0.0)	-

*No *An. funestus* were collected in the non-irrigated villages.

### Impact of canal water release on vector abundance

**Figure 4:**
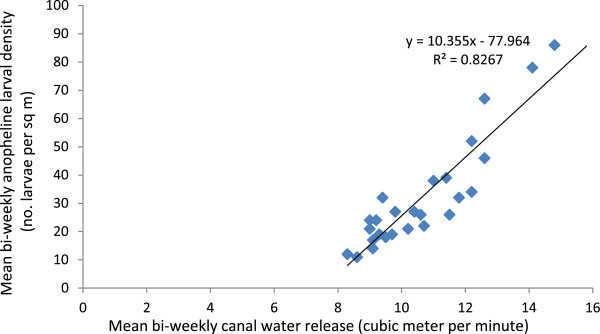
**Scatter graph showing a strong positive correlation between mean bi-weekly anopheline larval density and mean bi-weekly canal water release in the irrigated villages in the Ziway area, Ethiopia, between November 2009 and October 2010.**

**Figure 5:**
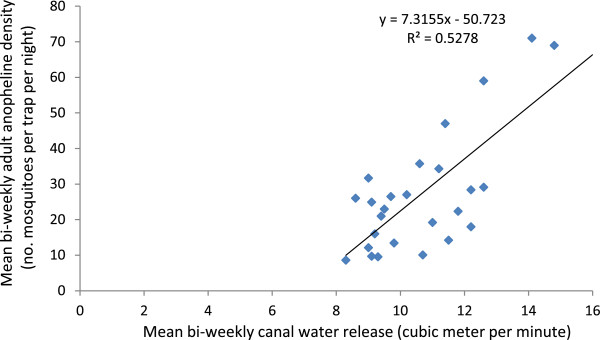
**Scatter graph showing a potential positive correlation between bi-weekly adult anopheline density and mean bi-weekly canal water release lagged by 2 weeks in the irrigated villages in the Ziway area, Ethiopia, between November 2009 and October 2010**.

Person’s correlation analysis revealed that bi-weekly anopheline larval densities were strongly positively correlated (r^2^ = 0.83; *P* < 0.05) with bi-weekly canal water releases in the irrigated study villages (Figure 4). This relationship was linear and it was also noted that one unit increase in canal water release could result in about a ten unit increase in anopheline larval density in the irrigated villages. Furthermore, bi-weekly anopheline larval densities lagged by two week were also positively correlated (r^2^ = 0.62; *P* < 0.05) with bi-weekly adult anopheline densities in the irrigated villages (Figure 5). Overall, these results indicate the strong link between irrigation-associated larval breeding and vector abundance in the irrigated villages.

**Figure 6:**
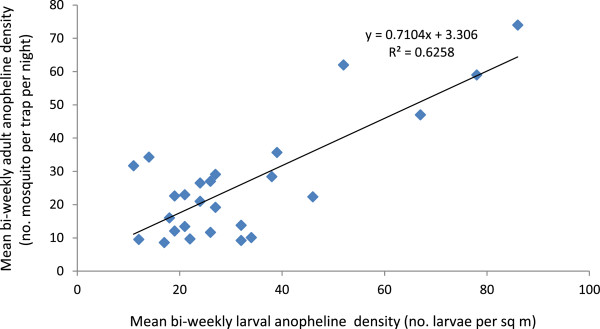
**Scatter diagram showing a potential positive correlation between mean bi-weekly larval anopheline density and mean bi-weekly adult anopheline density lagged by 2 weeks in the irrigated villages in the Ziway area, Ethiopia, between November 2009 and October 2010.**

In addition, Person’s correlation analysis indicated that mean bi-weekly adult anopheline density was positively correlated (r^2^ = 0.53; *P* < 0.05) with mean bi-weekly canal water releases lagged by two weeks in the irrigated villages (Figure 6). Furthermore, the anopheline larval breeding surface area in the irrigated villages (68 sq m) was four-time larger than the non-irrigated villages (12 sq m) during the study period.

### Impact of vector abundance on malaria incidence

**Figure 7:**
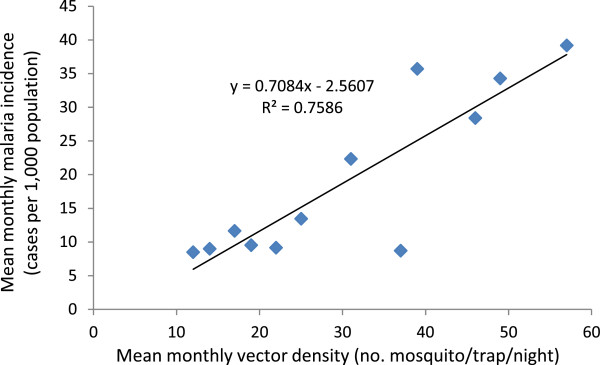
**Scatter graph showing a positive correlation between monthly malaria incidence and monthly vector density lagged by one month in the irrigated villages in the Ziway area, Ethiopia, between November 2009 and October 2010.**

Correlation analysis indicates that monthly malaria incidences (cases per 1,000 population) were significantly correlated (r^2^ = 0.76, *P* < 0.05) with monthly malaria vector density (i.e., *An. arabiensis* and *An. pharoensis*) lagged by one month in the irrigated villages (Figure 7). This implies that higher vector density in any one month was associated with higher malaria incidence in the following month. This suggests that an increase in vector density could lead to an increase to malaria transmission. In contrast, monthly malaria incidence in the non-irrigated villages, was not significantly correlated (r^2^ = 0.21, *P* > 0.05) with monthly vector densities lagged by one month during the study period.

## Discussion

The present study indicated that the small-scale irrigation schemes of Central Ethiopia resulted in increased malaria transmission in communities living in the irrigated villages throughout most of the year. Irrigation-associated mosquito-breeding habitats (irrigated field puddles, leakages from irrigation canals and non-functional canal pools) were the prominent malaria vector breeding sites throughout the year. The high vector densities of *An. arabiensis* and *An. pharoensis* in the irrigated villages coupled with their potential to inoculate 27.3 and 7.5 *P. falciparum* infective bites per person per year, respectively, confirms the significant risk of malaria transmission in communities living close to the irrigation schemes. Moreover, due to the availability of water for mosquito breeding, the irrigation schemes appear to extend the period of malaria transmission into the dry season in the irrigated villages. These findings were consistent with previous findings elsewhere in Ethiopia [6, 9, 23, 29]. In contrast to the present study findings, rice irrigated villages in the Lower Moshi irrigation scheme in northern Tanzania [30] and the *Office du Niger* irrigation scheme of Mali [15, 31] significantly increased vector densities, but without amplifying the EIR in the irrigated villages, while the EIR rose in the non-irrigated villages. The explanation for the observed higher vector density but a lower EIR in the irrigated villages than the non-irrigated villages was due to lower vectorial capacity as a consequence of amplified vector densities competing for human blood feeding.

Significantly higher monthly malaria prevalence coupled with higher EIR in the irrigated villages than the non-irrigated villages in the present study suggest the risk of malaria transmission in the irrigated villages throughout most of the year. In Western Ethiopia, Jaleta *et al.* [29] reported a 5.7-fold higher annual EIR by *An. arabiensis* in irrigated sugarcane villages than non-irrigated villages. The present study confirmed that dry season malaria transmission was established in the irrigated villages of Central Ethiopia. In tropical Africa where seasonality in rainfall drives the seasonal dynamics of malaria transmission, the presence of irrigation activities has been shown to have a dampening effect on the seasonality of malaria transmission [32]. A previous study indicated that rainfall is the main climatic determinant of seasonal malaria transmission in the Ziway area [33]. Such seasonal transmission is common in the Ethiopian Rift Valley [9, 34–36]. However, the present study confirmed that the irrigation schemes in the Ziway area have created conducive conditions for mosquitoes breeding during the dry season and thus the period of malaria transmission is extending throughout most of the year. Significantly higher larval anopheline densities observed in the irrigated villages than in the non-irrigated villages throughout the study period confirms that irrigation drives mosquito abundance. Similar results were previously reported in the same study area [9], Northern Ethiopia [23], and Western Ethiopia [29]. Furthermore, the present study confirmed the presence of malaria sporozoite-infected mosquitoes during the dry season. Similar findings were reported from irrigated sugarcane farms in Western Ethiopia where higher sporozoite rates were observed throughout the months of the year in irrigated villages than the non-irrigated villages [29]. Likewise, irrigation schemes in semi-arid areas of Sahel in Mali were blamed for extending malaria transmission into the dry season [15, 37].

Strong positive correlation between canal water release and anopheline larval density coupled with the presence of leakage pools from irrigating canals and water-logged field puddles in the irrigated villages indicate improper canal water management that led to increased malaria vector breeding and thus intensified malaria transmission. This is due to the thriving of the two malaria vector species (*An. arabiensis* and *An. pharoensis*) in irrigated fields and seepages created by the irrigation schemes. Similar findings were previously reported for these two malaria vectors from the Ziway area of Ethiopia [9] and the Mwea irrigation scheme of Kenya [38, 39]. In the northern Ethiopia, proper environmental management around irrigation microdams was reported to significantly reduce vector density (by 49%) [23].

The present study indicated that *An. arabiensis* predominantly feeds indoors while *An. pharoensis* tends to feed more outdoors than indoors in the study area. Similar findings were reported from the Koka area of Central Ethiopia, some 60 km north of the present study area [9]. The outdoor feeding habit of these malaria vector species is worrisome as the major vector control measure advocated in Ethiopia is insecticide-treated bed nets, which are largely effective for indoor feeding vectors. Moreover, most farmers in the irrigated villages tend to stay outside working on their farmlands during the early hours of the evening (personal observation), and thus are highly exposed to infective bites from exophagic vector mosquitoes. Studies in Ethiopia indicated that the peak biting activity of these two vector species occurs early in the evening before people retire to bed, thus compromising the efficacy of bed nets [9, 40]. This suggests the need for additional vector control measures such as water management options, especially in areas where water resources development schemes are constructed.

The high HBI observed in *An. arabiensis* both in the irrigated (0.80) and non-irrigated (0.73) villages coupled with the presence of *P. falciparum* sporozoite-infected *An. arabiensis* reaffirms the important role of this species in malaria transmission in Central Ethiopia. Comparable HBI results were previously documented for this species in the present study area (0.78) [9], in the adjacent Koka area (0.76) [36] and in northern Ethiopia (0.73) [23]. In contrast, lower HBI (0.1-0.5) results were reported for this species in Tanzania [41] and Kenya [17]. This strong zoophilic tendency of *An. arabiensis* might be the result of differences in species strains and high presence of cattle readily available for mosquito blood meal in these areas. The sporozoite rate (1.67%) reported for this species in the present study for the irrigated villages was comparable to results from previous studies in same study area (1.18%) [9]. On the other hand, *An. pharoensis* (HBI = 0.64) was found predominantly feeding outdoors on human blood with *P. falciparum* sporozoite infection that confirmed its role in malaria transmission. The role of this species in malaria transmission was previously confirmed in Central Ethiopia [9, 36]. *Anopheles coustani* and *An. funestus* were exophagic and primarily zoophilic with no role in malaria transmission. Similar findings were previously documented from the same study area [20], in the neighbouring Koka area [35] and northern part of Ethiopia [23]. Despite significantly higher vector densities in the irrigated villages, the difference in HBI between the irrigated and non-irrigated villages was not significant. Ijumba and Lindsay [7] pointed out that water resources development projects may cause a shift towards less endophilic and anthropophilic malaria vectors. However, the scope of the present study is too limited to comment on this.

A strong positive correlation between canal water releases and larval/adult vector densities clearly indicates the potential of using canal water management for malaria vector control around irrigation schemes. Mutero *et al.* [11] found that intermittent irrigation in the Mwea rice irrigation scheme of Kenya resulted in lower mosquito larval densities and survival. The present study indicated that higher canal water releases were associated with higher anopheline larval density, and higher vector abundance in the following fortnight. The two-week lag is explained by the fact that aquatic stages of mosquitoes require at least two weeks (in ambient temperatures, >21°C) to complete their aquatic life cycle and emerge as adult mosquitoes [42]. As the larval mosquitoes could complete their aquatic development in two-week period, no lag period was applied for bi-weekly canal water releases. The present study found that a unit increase in canal water releases could lead to a ten unit increase in anopheline larval density – suggesting the potential of proper canal water management for malaria vector control. During harvesting months of the year (December – January and May – June), low anopheline larval densities were observed as a consequence of low canal water releases (Figure 2). Furthermore, if proper water management is employed to minimize unwanted water logging, it would substantially reduce vector abundance and hence malaria transmission. Similarly, in the Mwea Irrigation Scheme of Kenya, Muturi *et al.* [39] suggested that proper water management could reduce vector breeding in irrigation-associated breeding sites, although the authors did not indicate what specific manipulations of the canal releases would bring about low vector breeding.

## Conclusion

This study revealed that poor canal water management led to a proliferation of malaria vector mosquito breeding sites and intensified malaria transmission in the irrigated villages in Central Ethiopia. Proper canal management by reducing water releases without affecting crop productions (only avoiding unnecessary water logging in the field) during the months of peak malaria transmission could reduce vector abundance and malaria transmission in the irrigated villages. Moreover, health education is needed to encourage irrigators to use personal protections for malaria prevention. The finding that the malaria incidence is associated with adult vector abundance and vector abundance is correlated with larval abundance, which is affected by canal water releases, reaffirms the importance of proper canal water management on malaria transmission. Field experimental studies are needed to further assess how manipulating canal water release could reduce larval and adult vector abundance and hence lower the risk of malaria transmission.

## Abbreviations

CDC: Center for disease control and prevention
ELISA: Enzyme-linked imminosorbant assay
CI: Confidence interval
DF: Degree of freedom
EIR: Entomological inoculation rate
HBI: Human blood index
US$: US dollar
WHO: World health organization.
